# Factors associated with influenza and COVID-19 vaccination among health workers in Lao PDR, 2023

**DOI:** 10.1016/j.vaccine.2025.127006

**Published:** 2025-03-14

**Authors:** Chansay Patthammavong, Natalie Wodniak, Kongxay Phounphenghack, Chankham Tengbriacheu, Bandith Soumphonphakdy, Viengkhan Phixay, Chansavang Vongkhamsao, Viengphone Khanthamaly, Jaymin C. Patel, Martha P. Montgomery, Margaret McCarron, William W. Davis, Julie Carlton, Eva S. Bazant, Ann C. Moen, Phonethipsavanh Nouanthong

**Affiliations:** a Lao Mother and Child Health Center, Ministry of Health, Vientiane, Laos; b Task Force for Global Health, Atlanta, GA, USA; c U.S. Centers for Disease Control and Prevention, Vientiane, Laos; d U.S. Centers for Disease Control and Prevention, Atlanta, GA, USA; e Thailand Ministry of Public Health-U.S. Centers for Disease Control and Prevention, Nonthaburi, Thailand; f Lao PDR National Immunization Technical Advisory Group, Vientiane, Laos

**Keywords:** Influenza, COVID-19, Vaccination, Lao PDR, Vaccine acceptance, Health workers

## Abstract

Understanding vaccine uptake and related factors among health care workers is critical to successful vaccination programs. A cross-sectional survey was conducted in central, provincial, district hospitals and health centers among health workers in Lao People’s Democratic Republic (PDR) in November 2023 to assess health workers’ experience with influenza and COVID-19 vaccination, vaccination uptake, intended uptake, and intention to recommend both vaccinations to patients in the future. Logistic regression was used to identify factors associated with these practices. Among 1228 surveyed health workers in six provinces, 55 % were nurses, assistant nurses, or midwives; 32 % were doctors or assistant doctors; and 14 % had other occupations. Overall, 77 % of respondents were female, and the median age was 34 years (interquartile range 29–42 years). Current influenza vaccination and receipt of COVID-19 booster doses were 70 % (95 % confidence interval [CI]: 62–78 %) and 90 % (95 % CI: 87–92 %), respectively. If vaccines were available for free in the future, approximately 94 % and 92 % of health workers would receive influenza and COVID-19 vaccination, respectively. Nearly all health workers would recommend influenza (98 %) and COVID-19 (95 %) vaccination to their patients. Health workers who had received influenza vaccination prior to the COVID-19 pandemic were more likely to have received current influenza vaccination (adjusted odds ratio [aOR], 95 % CI: 3.7, 2.8–4.9) and to intend to receive influenza vaccination in the future (aOR 2.7, 95 % CI: 1.1–6.8). Health workers who were vaccinated for influenza in the current season were more likely to receive COVID-19 booster doses and to intend to receive future booster doses (aOR, 95 % CI: 2.2, 1.3–3.7 and 2.5, 1.2–5.1, respectively). Intention to recommend influenza vaccination to patients was associated with the intention to recommend COVID-19 vaccination to patients, and vice versa. High acceptance for influenza and COVID-19 vaccination among health workers can support a successful vaccination program in Lao PDR.

## 1. Introduction

Vaccines for seasonal influenza and COVID-19 can reduce disease severity and lessen the burden on health systems. As COVID-19 becomes endemic and the threat of seasonal influenza continues, it is important to understand the drivers of uptake and hesitancy for these vaccines in local contexts. Vaccination acceptance among health workers is particularly important because of health workers’ susceptibility for infection, potential for spreading the viruses to patients, and for reassuring the general population that vaccines are safe and effective. Globally, cross-sectional surveys have been used to assess vaccination uptake and hesitancy and to inform vaccination programming [1–5].

Since 2012, Lao People’s Democratic Republic (PDR) has maintained a seasonal influenza vaccination program for high priority populations including health workers, older adults, pregnant people, and adults with chronic conditions [6–8]. Lao PDR also successfully initiated pandemic vaccination rollouts, both during the 2009 H1N1 influenza pandemic and during the COVID-19 pandemic. Although both influenza and COVID-19 vaccinations are recommended for priority groups including health workers in Lao PDR, neither vaccination is mandated. Influenza circulates year-round in Lao PDR with the primary peak occurring between July and December and most vaccinations occurring from April to June [8]. Influenza seasonal vaccination coverage has been high among health workers [7,9]; however, the supply of vaccines remains insufficient to achieve high coverage among pregnant people and older adults with chronic conditions [8]. In 2020, there were 39,502 influenza vaccine doses administered in Lao PDR, with over half given to health workers.

COVID-19 vaccine rollout began in April 2021, through the National Deployment and Vaccination Plan [10,11]. Uptake of COVID-19 vaccination is high in Lao PDR: as of May 2024, 86.5 % of the population had received at least one COVID-19 vaccine dose and 78.0 % had completed a primary series, with 98.8 % of health workers having completed a primary series. In 2023, the Ministry of Health began to integrate COVID-19 vaccination into routine immunization programs.

In November 2023, a knowledge, attitudes, and practices survey was conducted among health workers in Lao PDR. The objectives of the survey were: 1) to understand the drivers of influenza and COVID-19 vaccination uptake among health workers, 2) to understand the influence of previous experience with seasonal influenza vaccination on the acceptance of COVID-19 vaccination among health workers, and 3) to understand the drivers of health workers recommending influenza and COVID-19 vaccination to their patients. This paper examines the factors associated with influenza and COVID-19 vaccination uptake and intended uptake among health workers in Lao PDR, and the intention to recommend both vaccinations to patients. Considering the challenges of influenza vaccine procurement and recent integration of influenza and COVID-19 vaccination policy, the findings from this survey will be used for strategic planning to ensure continued success of these vaccination programs.

## 2. Methods

### 2.1. Study design

A cross-sectional, paper-based survey was conducted among health workers in Lao PDR during two weeks in November 2023. The survey was structured using a standard protocol and questionnaire based on the Health Belief Model to measure perceptions of susceptibility and severity of influenza disease and the benefits of, barriers to, and motivators for vaccination. [12] The questionnaire was adapted based on discussions with vaccination experts in Lao PDR and translated into the Lao language. The survey collected information on demographic characteristics, knowledge and attitudes towards influenza and COVID-19, uptake and intended uptake of influenza and COVID-19 vaccination, and recommendation of influenza and COVID-19 vaccination to patients.

### 2.2. Participants

Health workers were defined as all staff working in health facilities. Priority was given to health workers in patient-facing roles, but those in non-patient-facing roles (e.g., laboratorian, administrative staff) were included as necessary to meet the target sample size.

### 2.3. Variables

Six outcomes were defined: 1) influenza vaccination uptake; 2) intended uptake of influenza vaccination in the future; 3) COVID-19 vaccination uptake; 4) intended uptake of annual COVID-19 boosters in the future; 5) intention to recommend influenza vaccination to patients in the future; and 6) intention to recommend COVID-19 vaccination to patients in the future. Outcomes were dichotomized, combining “No” and “Don’t know/not sure” into a single category. Predictors included ever having examined or diagnosed patients with influenza or COVID-19, ever having treated severe cases of influenza or COVID-19, receipt of influenza vaccination prior to the COVID-19 pandemic and in the last season, receipt of COVID-19 primary series or booster doses, intention to receive influenza and COVID-19 vaccination in the future, and intention to recommend influenza and COVID-19 vaccination to patients in the future. Potential confounders included age (continuous in 10-year bands), sex, occupation, health facility level, years worked in a healthcare profession (continuous), and self-reported presence of any underlying conditions (including obesity, diabetes, heart disease, lung disease, immunocompromising condition, and other conditions).

### 2.4. Data sources

Questionnaire responses were recorded on paper forms and transcribed into an electronic database (Kobo Toolbox, https://www.kobotoolbox.org/) at the end of each day of data collection. Entered data was checked for accuracy once by data collection teams and validated in the electronic database.

Ethical approval was obtained from the Lao PDR National Ethics Committee for Health Research (#2023–24) in November 2023. This project was reviewed by the U.S. Centers for Disease Control and Prevention (CDC) and was conducted consistent with applicable federal law and CDC policy (e.g., 45 CFR 46.102(l)).

### 2.5. Sampling methods

**Fig. 1.**
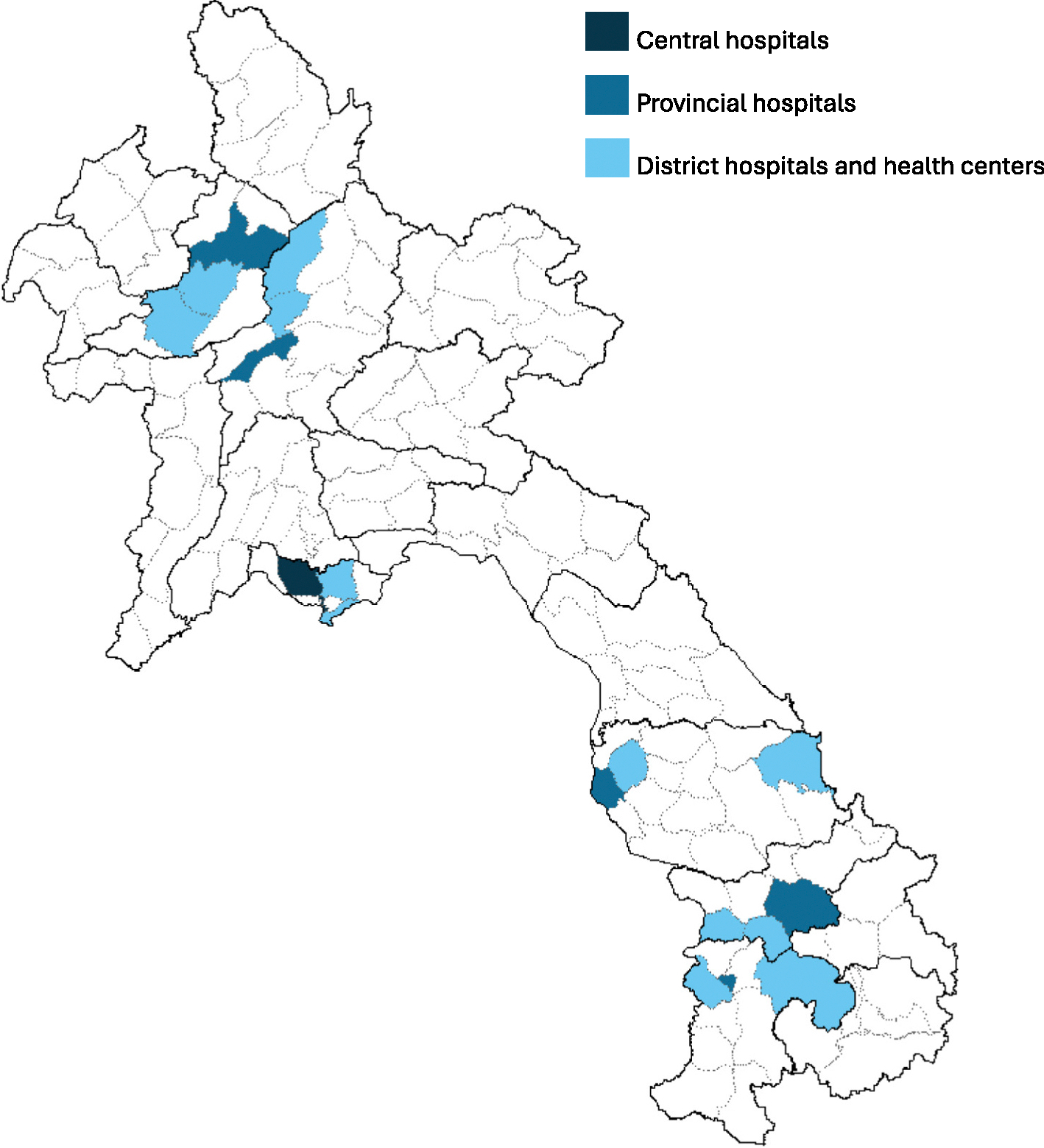
Map of districts with hospitals and health centers participating in health care worker survey—Lao PDR, November 2023. Provincial borders are shown by solid lines. District borders are shown by dotted lines. Shape file data obtained from data.opendevelopmentmekong.net.

Six provinces were selected based on the existence of influenza sentinel surveillance systems, representing all geographic areas in Lao PDR (northern, central, and southern regions). In each province, health workers were recruited from the provincial hospital (highest level with one in each province), district hospitals (middle level hospital), and health centers (lower-level outpatient clinics), and also from central hospitals in Vientiane. A map of selected districts is shown in the Fig. 1.

In each of the six provinces, two districts were randomly selected, and two health centers were randomly selected from each district. A resulting total of one provincial hospital, two district hospitals, and four health centers were included in each province. In Vientiane capital, six central hospitals were selected out of a total of seven central hospitals. The seventh central hospital was excluded from the study as it was used for training during the survey development process. Each selected hospital provided the study team with the total number of health workers on its roster to assist with sampling estimations.

In each health facility, the target number of health workers were randomly sampled from the roster of all health workers in the facility. Selected health workers were approached in person to provide, written, informed consent to participate in the survey. If the selected health worker was not available, survey teams attempted to contact them the next day, and otherwise randomly selected another health worker from the roster. Non-response to the survey was not recorded, but staff conducting the survey reported that non-response was uncommon.

A baseline sample size (n) of 385 was calculated with the following formula, based on a desired precision (e) of 0.05 and a total target population (N) of 10,000 health workers:


$$n=\frac{N}{1\;+\;N{\left(e\right)}^{2}}$$

Design effect was used to account for the cluster-based design, with provinces designated as the sampling clusters. A design effect of 4.75 was calculated by $\text{DE}=1+\text{m}\alpha^{2}\mu$, assuming a mean cluster size (m) of 30 participants, intra-class correlation (α) of 0.5, and an outcome prevalence (μ) of 0.5. With the design effect, a target sample size of 1827 was calculated. After consultation with the Ministry of Health, the target sample size was reduced to 1221 for feasibility reasons and to better reflect the size of health facilities and number of health workers in selected provinces. This sample size gives a calculated precision (e) of 0.062.

The target sample size for each type of health facility is shown in Table 1.

### 2.6. Statistical methods

The analysis accounted for the multi-stage survey design. The primary sampling unit was defined as health facility, and provinces were set as clusters. Sampling weights were calculated as the probability of selection from two stages of sampling within each province:

- Selection of the health facility: p_1_ = (n_1_/N_1_), where n_1_ = the number of district or health center facilities selected for the survey in each province, and N_1_ = the total number of districts or health facilities in the province
- Selection of the health worker: p_2_ = (n_2_/N_2_), where n_2_ = the number of health workers selected in each facility, and N_2_ = the total number of health workers within the facility
- Sampling probability: P = p_1_*p_2_
- Sampling weight: 1/P

Demographic variables were described as frequency and percent for categorical variables, and median and interquartile range (IQR) for numeric variables. For questions relating to influenza and COVID-19 vaccination, frequency, survey proportions, and Normal (Wald) 95 % confidence intervals (CI) were described.

Logistic regressions were performed to determine factors associated with the six primary outcomes. Variables including age and years worked in the healthcare profession were assessed as continuous variables or categorical in bivariate logistic regressions and compared using the Hosmer-Lemeshow goodness-of-fit test [13].

Bivariate logistic regression was performed to assess the factors associated with each of the outcomes. Variables with a significance of *p* < 0.2 in bivariate models were considered for inclusion into multivariable models. Multivariable models were adjusted for age and sex a priori and were fit using hierarchical stepwise selection [14], with variables with a significance of *p* < 0.05 retained in final models. Odds ratios, adjusted odds ratios (aORs), and their respective 95 % CIs were reported. Collinearity within multivariable models was assessed by calculating the variance inflation factor (VIF), with VIF ≥ 2.5 indicating collinearity [15]. Complete case analysis was conducted, and observations missing values for any of the analysis variables were discarded.

All analyses were conducted in Stata SE 18.0 (StataCorp. 2023. Stata Statistical Software: Release 18. College Station, TX: StataCorp LLC). The map figure was created using R Statistical Software (v4.4.1) with packages sf, ggplot2, readxl, and dplyr [16]. Shape file data were obtained from Open Development Laos [17].

## 3. Results

A total of 1228 health workers were interviewed and included in the analysis, with 184 (15.0 %) from central hospitals, 504 (41.0 %) from provincial hospitals, 419 (34.1 %) from district hospitals, and 121 (9.9 %) from health centers. Participants were mostly female (77.4 %), an average of 36 years old, and were predominantly nurses (43.7 %) or doctors (26.3 %). Overall, 1089 (88.2 %) of participants worked in a patient-facing role (Table 2).

### 3.1. Influenza

Most health workers correctly believed that Lao PDR has an influenza vaccination program for either health workers or populations at risk of severe outcomes, and 66.7 % (95 % CI: 61.7 %–71.8 %) believed they could receive an influenza vaccination if they wanted to. One-half (53.1 %, 95 % CI: 47.2 %–59.0 %) had ever examined or diagnosed a patient with influenza, and 42.7 % (95 % CI: 36.5 %–48.9 %) had treated a severe or life-threatening case of influenza. An estimated 43.4 % (95 % CI: 36.9 %–48.9 %) of health workers incorrectly believed that the influenza vaccine can protect against SARS-CoV-2 infection (Table 3). Correct responses to this question (influenza vaccine does not protect against SARS-CoV-2 infection) were < 50 % correct for all occupations.

Influenza vaccination uptake in the last season was 69.5 % (95 % CI: 61.6 %–77.5 %). Nearly all health workers (94.4 %, 95 % CI: 91.2 %–97.7 %) indicated they would receive the influenza vaccination in the future if available to them and offered for free, and 98.3 % (95 % CI: 97.3 %–99.2 %) indicated they would recommend vaccination to their patients if it were available (Table 4). Among 273 health workers who were not vaccinated in the last season, the most common reasons were that they did not have time to get vaccinated (30.3 %, 95 % CI: 20.6 %–42.1 %) or that they were not aware that the vaccine was available to health workers (22.1 %, 95 % CI: 15.0 %–31.4 %).

### 3.2. COVID-19

An estimated 61.4 % (95 % CI: 56.0 %–66.8 %) of health workers had ever examined or diagnosed a patient with COVID-19, with 43.9 % (95 % CI: 38.3 %–49.6 %) having treated life-threatening cases of COVID-19. An estimated 36.2 % (95 % CI: 31.4 %–41.1 %) of health workers incorrectly believed that the COVID-19 vaccine can protect against influenza virus infection (Table 3). Correct responses to this question (COVID-19 vaccine does not protect against influenza infection) were < 50 % correct for all occupations. Of those who believed COVID-19 vaccines could prevent influenza, 70.6 % (322/456) also believed that influenza vaccines could prevent COVID-19.

COVID-19 vaccination uptake was high, with 98.6 % (95 % CI: 97.5 %–99.2 %) having received any doses, 96.6 % (95 % CI: 94.6 %–97.9 %) having completed a primary series, and 90.0 % (95 % CI: 87.4 %–92.2 %) having received one or more booster doses. The intention to continue to receive annual booster doses was also high at 91.6 % of health workers (95 % CI: 89.2 %–93.5 %). Nearly all health workers recommended COVID-19 vaccination to patients during the pandemic and currently recommend them, and 95.4 % (95 % CI: 93.9 %–96.6 %) intended to continue to recommend COVID-19 vaccination to patients in the future if it becomes an annually recommended vaccine (Table 5).

### 3.3. Factors associated with influenza vaccination uptake and intended uptake

In the fully adjusted model, receipt of influenza vaccination prior to the start of the COVID-19 pandemic (aOR: 3.7, 95 % CI: 2.8–4.9), receipt of at least one COVID-19 booster dose (aOR: 2.5, 95 % CI: 1.5–4.2), and increasing age (aOR: 1.4, 95 % CI: 1.1–1.7) were associated with receipt of influenza vaccination in the last season.

Several covariates were associated with the intention to receive influenza vaccination in the future if they are available to health workers. After adjusting for age and sex, health workers in district hospitals when compared to central hospitals had greater odds of reporting intention to receive influenza vaccination in the future, (aOR: 4.7, 95 % CI: 1.4–15.3). Health workers who had ever examined or diagnosed a patient with influenza (aOR: 2.7, 95 % CI: 1.6–4.7), received influenza vaccination prior to the start of the COVID-19 pandemic (aOR: 2.7, 95 % CI: 1.1–6.8), or intended to receive annual COVID-19 vaccination in the future (aOR: 9.6, 95 % CI: 4.4–21.3) also had higher odds of intended influenza vaccination uptake (Table 6).

### 3.4. Factors associated with COVID-19 vaccination uptake and intended uptake

Receipt of an influenza vaccination in the last season (aOR: 2.2, 95 % CI: 1.3–3.7) and increasing age (aOR: 1.9, 95 % CI: 1.4–2.7) were associated with receipt of one or more COVID-19 booster doses, after adjusting for sex.

Occupation, health facility level, and receipt of influenza vaccination in the last season were associated with the intention to receive COVID-19 booster doses annually if recommended by the WHO. When compared to doctors, assistant doctors (aOR: 4.9, 95 % CI: 1.5–15.8), midwives (aOR: 3.1, 95 % CI: 1.4–6.9), and those with other health care occupations (aOR: 2.6, 95 % CI: 1.2–5.9) had greater odds of intended COVID-19 vaccination uptake, while assistant nurses (aOR: 0.4, 95 % CI: 0.2–0.8) had lower odds of intended uptake. Health workers in district hospitals (aOR: 2.2, 95 % CI: 1.5–3.2) and health centers (aOR: 5.2, 95 % CI: 1.5–18.9) had greater odds of intended vaccination uptake compared to health workers in central hospitals. Receipt of influenza vaccination in the last season was also associated with intended COVID-19 vaccination uptake (aOR: 2.5, 95 % CI: 1.2–5.1; Table 7).

### 3.5. Factors associated with the recommendation of influenza and COVID-19 vaccination to patients

Ever having examined or diagnosed a patient with influenza (aOR: 6.7, 95 % CI: 1.7–26.0) and the intention to recommend COVID-19 vaccination to patients in the future (aOR: 21.4, 5.3–85.9), were associated with the intention to recommend influenza vaccination to patients in the future if vaccination is offered and available, after adjusting for age and sex (Table 8).

Intention to receive annual COVID-19 booster doses (aOR: 24.7, 95 % CI: 11.4–53.5) and the intention to recommend influenza vaccination to patients if they were made available in the future (aOR: 21.1, 95 % CI: 5.3–84.3) were associated with the intention to recommend annual COVID-19 vaccination to patients, after adjusting for age and sex (Table 9).

## 4. Discussion

Overall, this survey demonstrates a high acceptance of influenza and COVID-19 vaccination among health workers, and a high propensity to recommend vaccination to patients. The challenge for Lao PDR in maintaining a sustainable influenza and COVID-19 vaccination program for health care workers will lie not with improving vaccination acceptance, but in ensuring affordable and sustainable vaccine supply.

Uptake of both influenza and COVID-19 vaccination were high among health workers, with 97 % having completed a COVID-19 primary series and 70 % having received an influenza vaccination in the last season. Lower vaccination coverage for influenza might be because of influenza vaccine supply shortages compared to COVID-19 vaccines in Lao PDR during the survey period. Only 50,000 influenza doses were obtained for health care workers and other recommended groups in 2023, short of the 200,000 projected doses that would be needed to cover 50 % of health workers and other recommended groups. [8] In 2024, the number of doses purchased decreased to 34,000 because of inflation. Advanced planning for vaccine procurement is crucial to meet vaccination targets. Lao PDR remains reliant on international support for the procurement of additional vaccine doses but has experienced a decline in international vaccine donations since 2023 [18]. Continued partnerships with international donors will be necessary to achieve targeted levels of vaccination coverage in the population. Future studies could also assess health worker willingness to pay for vaccination and other innovative solutions for sustainable financing. [19,20]

Acceptance of both vaccines is high, with 94 % of health workers reporting that they would receive influenza vaccination in the future if vaccines were available and offered for free, and 92 % of health workers reporting they would receive annual COVID-19 boosters if recommended by the WHO. The high willingness of health workers in Lao PDR to receive future doses of COVID-19 vaccines surpasses survey results seen in other countries [21–23], which may be related to high overall vaccination uptake among health workers in Lao PDR, as exemplified by a 2016 health worker survey on routine immunizations [24]. This survey also showed that health workers intend to recommend both vaccinations to patients in the future if vaccines are available and recommended by the WHO, with 98 % and 95 % intending to recommend influenza and COVID-19 vaccines, respectively.

Health workers could benefit from continued trainings on influenza and COVID-19 vaccination, as several gaps in knowledge were identified. Notably, a substantial proportion of health workers believed that influenza vaccines could prevent SARS-CoV-2 infection, and that COVID-19 vaccines could prevent influenza virus infection. These misunderstandings were present across all occupation groups. Outreach to health workers could provide clearer messaging that influenza vaccination protects against influenza and COVID-19 vaccination protects against COVID-19.

Multivariable logistic regression results showed significant associations between the uptake of influenza and COVID-19 vaccination, and between the intention to receive influenza vaccination and COVID-19 vaccination in the future, although several findings had notably wide CIs. These results indicate that vaccination behaviors may be greatly influenced by current practices regarding vaccination, and that remaining up to date on vaccinations including seasonal influenza vaccination may influence the acceptance of other vaccines, including those developed during future pandemics. Notably, health workers in district hospitals and health centers had greater intention to receive annual influenza and COVID-19 vaccination in the future, compared to health workers in central hospitals. This finding was unexpected and could be explored in future surveys. These results indicate that health workers in central hospitals may benefit from targeted education campaigns emphasizing the importance of health worker vaccination and their increased risk of infection due to contact with ill patients.

This study is subject to limitations. First, the calculated sample size was reduced based on logistical constraints and limited resources, and therefore, the power of this analysis may not have been sufficiently strong to capture all associations. Some findings, although statistically significant, had wide CIs suggesting uncertainty in the estimate. Second, although the initial protocol was written to include only health workers in patient-facing roles, health workers in non-patient-facing roles were included to meet the reduced sample size but represented only 12 % of participants. Results may have differed if only patient-facing health workers were included in the study. Third, this study is subject to common biases in survey analyses, including self-reported vaccination status. Fourth, several variables included in regression analyses had very small reference groups (including small proportions of health workers who would not recommend influenza or COVID-19 vaccination). Small reference groups may increase the likelihood that significant associations were discovered by chance. Finally, since this study was conducted in provinces with existing influenza sentinel surveillance programs, the results may not be generalizable to all health workers in Lao PDR, particularly those who work in other provinces.

## 5. Conclusion

This analysis indicates a high level of acceptance for influenza and COVID-19 vaccinations and emphasizes the need to identify a model of affordable and sustainable vaccine supply in Lao PDR. Previous experience with influenza vaccination was associated with both influenza and COVID-19 vaccination uptake and intended uptake, indicating that acceptance of routine or seasonal immunizations may influence vaccination acceptance in a pandemic situation. Results from this analysis can be used to inform messaging and the procurement of vaccines to meet the needs of health workers and other priority populations in Lao PDR.

## Figures and Tables

**Table 1:** Target sample size.

Health facilities	No. of sites	No. of health workers sampled per site	Target sample size

Central hospitals (Vientiane capital)	6	30	180
Provincial hospitals (1 per province, excluding Vientiane capital)	5	100	500
District hospitals (2 per province)	12	30*	420
Health centers (2 per district)	24	All staff	121
**Total**			**1221**

* 2 district hospitals with *n* = 40 and 2 district hospitals with *n* = 50, based on larger size of the health facilities.

**Table 2:** Demographic characteristics of health worker survey participants (*N* = 1228)—Lao PDR, November 2023.

Variable	n (%)

**Sex**	
Female	951 (77.4 %)
Male	274 (22.3 %)
Prefer not to say	3 (0.2 %)
**Age, years (median (IQR))**	34 (29–42)
**Years worked in the health sector (median (IQR))**	10 (5–17)
**Occupation**	
Nurse	536 (43.7 %)
Doctor	322 (26.3 %)
Midwife	130 (10.6 %)
Assistant doctor	66 (5.4 %)
Assistant nurse	7 (0.6 %)
Other*	165 (13.5 %)
**Presence of any long-term/chronic condition**	
Yes	178 (14.5 %)
No	1047 (85.3 %)
*Missing data*	3 (0.2 %)
**Type of chronic condition (*N* = 178)** ^†^	
Diabetes	63 (35.4 %)
Lung disease	38 (21.3 %)
Obesity	36 (20.2 %)
Immunocompromised	35 (19.7 %)
Heart disease	29 (16.3 %)
Other long-term condition	62 (34.8 %)
**Ward/Department** ^†^	
General/internal medicine	396 (32.2 %)
Obstetrics/gynecology	225 (18.3 %)
Emergency department	224 (18.2 %)
Pediatrics	163 (13.3 %)
Adult intensive care unit	83 (6.8 %)
Neonatal intensive care unit	49 (4.0 %)
Surgery	48 (3.9 %)
Radiology	27 (2.2 %)
Other	412 (33.6 %)
*Missing data*	8 (0.7 %)
**Type of patients treated** ^†^	
Pregnant people	666 (54.2 %)
Children	663 (54.0 %)
Adults with chronic diseases	629 (51.2 %)
Older adults (above 65)	592 (48.2 %)
Adults with infectious diseases	530 (43.2 %)
Other	281 (22.9 %)
*Missing data*	8 (0.7 %)

Abbreviations: IQR, interquartile range

* Other occupations include 137 participants in non-patient-facing roles (eg, administration, pharmacist, laboratorian) and 28 participants in patient-facing roles (eg, dentist, physical therapist)

† Participants could select more than one response

**Table 3:** Knowledge of and clinical experience with influenza and COVID-19 among health workers—Lao PDR, November 2023.

Indicator		n	Survey proportion (%)	Lower 95 % CI	Upper 95 % CI

**Influenza questions**					
Does Lao PDR have an influenza vaccination program? (*N* = 1225)	Yes, for health workers only	632	46.5 %	36.1 %	57.0 %
	Yes, for high-risk populations only	483	43.5 %	35.7 %	51.4 %
	No	24	2.1 %	0.9 %	3.3 %
	Unsure	86	7.7 %	4.7 %	10.7 %
Are you able to receive an influenza vaccine if you want to? (*N* = 1224)	Yes	844	66.7 %	61.7 %	71.8 %
	No	222	19.3 %	14.4 %	24.2 %
	Unsure	158	13.7 %	8.7 %	18.7 %
Have you ever examined or diagnosed a patient with confirmed seasonal influenza? (*N* = 1217)	Yes	629	53.1 %	47.2 %	59.0 %
	No	471	37.2 %	30.6 %	43.8 %
	I don’t remember	117	8.8 %	5.8 %	11.8 %
Have you ever treated a patient who developed a life-threatening condition due to influenza infection? (*N* = 1213)	Yes	501	42.7 %	36.5 %	48.9 %
	No	609	48.3 %	42.0 %	54.6 %
	I don’t remember	103	8.0 %	5.8 %	10.2 %
Can the influenza vaccine protect you from COVID-19? (N = 1224)	Yes	534	43.4 %	36.9 %	50.0 %
	No	530	41.4 %	34.1 %	48.7 %
	I don’t know	160	15.0 %	10.2 %	19.8 %
Do you remember the H1N1 influenza pandemic in 2009? (*N* = 1226)	Yes	512	43.8 %	38.8 %	48.8 %
	No	714	56.2 %	51.2 %	61.1 %
**COVID-19 questions**					
Have you ever examined or diagnosed a patient with confirmed COVID-19? (N = 1226)	Yes	783	61.4 %	56.0 %	66.8 %
	No	420	36.2 %	30.3 %	42.1 %
	I don’t remember	23	2.2 %	0.7 %	3.7 %
Have you ever treated a patient who developed a life-threatening complication due to COVID-19? (*N* = 1227)	Yes	569	43.9 %	38.3 %	49.6 %
	No	628	53.8 %	47.7 %	60.0 %
	I don’t remember	30	2.2 %	1.0 %	3.5 %
Can the COVID-19 vaccine protect you against influenza infection? (*N* = 1221)	Yes	456	36.2 %	31.4 %	41.1 %
	No	481	40.1 %	35.6 %	44.7 %
	I don’t know	284	23.0 %	19.1 %	26.9 %

**Table 4:** Influenza vaccination uptake and recommendation among health workers—Lao PDR, November 2023.

Indicator		n	Survey proportion (%)	Lower 95 % CI	Upper 95 % CI

Were you vaccinated during the 2009 H1N1 pandemic? (*N* = 503*)	Yes	370	73.7 %	67.0 %	80.4 %
	No	91	17.6 %	12.1 %	23.2 %
	I don’t remember	42	6.8 %	2.5 %	11.2 %
Did you ever receive an influenza vaccine prior to the start of the COVID-19 pandemic? (*N* = 1226)	Yes	836	69.4 %	64.7 %	74.1 %
	No	174	14.8 %	11.1 %	18.5 %
	I don’t remember	216	15.8 %	11.6 %	19.9 %
Did you receive an influenza vaccine in the last season? (N = 1225)	Yes	853	69.5 %	61.6 %	77.5 %
	No	273	23.2 %	16.2 %	30.3 %
	I don’t remember	99	7.2 %	5.1 %	9.2 %
If influenza vaccine was available to health workers and offered to you for free, would you receive it? (N = 1228)	Yes	1173	94.4 %	91.2 %	97.7 %
	No	30	2.9 %	1.5 %	4.3 %
	I don’t know	25	2.7 %	0.5 %	4.8 %
If influenza vaccine was available for your patients, would you recommend they get vaccinated? (*N* = 1228)	Yes	1208	98.3 %	97.3 %	99.2 %
	No	6	0.5 %	0.0 %	1.0 %
	I don’t know	14	1.3 %	0.5 %	2.0 %

* Among 503 respondents who remembered the 2009 H1N1 pandemic.

**Table 5:** COVID-19 vaccination uptake and recommendation among health workers—Lao PDR, November 2023.

Indicator		n	Survey proportion (%)	Lower 95 % CI	Upper 95 % CI

Have you received a COVID-19 vaccine? (N = 1226)	Yes	1206	98.6 %	97.5 %	99.2 %
	No	12	0.9 %	0.4 %	2.1 %
	I don’t remember	8	0.5 %	0.2 %	1.2 %
Did you complete a COVID-19 vaccine primary series? (N = 1226)	Yes	1171	96.6 %	94.6 %	97.9 %
	No	38	2.8 %	1.6 %	4.8 %
	I don’t know	17	0.6 %	0.3 %	1.3 %
Have you received any COVID-19 booster doses? (N = 1225)	Yes	1082	90.0 %	87.4 %	92.2 %
	No	133	9.6 %	7.5 %	12.2 %
	I don’t know	10	0.2 %	0.1 %	0.7 %
If yes, how many booster doses have you received? (*N* = 1063)	1	490	41.9 %	36.3 %	47.7 %
	2	466	47.8 %	40.9 %	54.8 %
	3	73	5.7 %	3.5 %	9.2 %
	4	31	2.6 %	1.3 %	5.2 %
	5	3	0.2 %	0.1 %	1.0 %
Will you receive booster doses annually if recommended by the WHO? (*N* = 1222)	Yes	1113	91.6 %	89.2 %	93.5 %
	No	57	3.9 %	3.1 %	4.8 %
	I don’t know	52	4.1 %	2.5 %	6.7 %
Did you recommend COVID-19 vaccines to patients during the pandemic? (N = 1222)	Yes	1210	98.3 %	96.6 %	99.2 %
	No	12	1.0 %	0.5 %	2.2 %
Do you recommend COVID-19 vaccines to patients now? (*N* = 1219)	Yes	1205	98.0 %	96.7 %	98.7 %
	No	14	1.3 %	0.7 %	2.3 %
If the COVID-19 vaccine becomes an annually recommended vaccine, will you recommend it to patients? (N = 1228)	Yes	1178	95.4 %	93.9 %	96.6 %
	No	28	2.5 %	1.7 %	3.6 %
	I don’t know	22	2.1 %	1.3 %	3.4 %

**Table 6:** Factors associated with influenza vaccination uptake and intended uptake among health workers—Lao PDR, November 2023.

Variable	Influenza vaccination uptake in the last season				Intention to receive influenza vaccination in the future if available and offered for free			
	
	OR	95 %CI	aOR (*N* = 1156)	95 % CI	OR	95 % CI	aOR (*N* = 1095)	95 % CI

Age (for each 10-year increase in age)	**1.59**	**1.29–1.98** *	**1.37**	**1.11–1.70**	1.03	0.70–1.52*	0.65	0.30–1.41
Female	1.59	0.97–2.61*	1.55	0.89–2.70	1.35	0.66–2.76*	1.30	0.34–5.03
Occupation								
Doctor	Ref.				Ref.			
Assistant doctor	0.66	0.29–1.48*			*Removed from model; perfectly predicts outcome*			
Nurse	**1.53**	**1.07–2.19** *			2.65	0.66–10.67*		
Assistant nurse	0.85	0.44–1.64*			**0.16**	**0.09–0.28** *		
Midwife	1.63	0.68–1.34*			4.55	0.60–34.44*		
Other	0.96	0.69–1.34*			1.84	0.50–6.73*		
Health facility level								
Central hospital	Ref.				Ref.			
Provincial hospital	1.39	0.79–2.45*			2.18	0.89–5.35*	2.41	0.72–8.10
District hospital	2.18	1.03–4.59*			**4.77**	**1.94–11.71** *	**4.68**	**1.43–15.29**
Health center	0.87	0.43–1.74*			*Removed from model; perfectly predicts outcome*			
Years in healthcare	**1.05**	**1.02–1.07** *			1	0.95–1.05		
Presence of any underlying chronic conditions^†^	1.43	0.86–2.39			0.53	0.24–1.17*		
Ever examined or diagnosed a patient with influenza	0.95	0.56–1.60			**2.45**	**1.70–3.52** *	**2.72**	**1.58–4.69**
Ever treated a patient with a life-threatening complication due to influenza	1.05	0.72–1.55			0.93	0.46–1.88		
Received influenza vaccine prior to start of COVID-19 pandemic	**4.08**	**3.27–5.08** *	**3.68**	**2.77–4.89**	**2.94**	**1.99–4.35** *	**2.70**	**1.07–6.83**
Encountered resistance or hesitancy from patients when recommending influenza vaccine	1.18	0.78–1.79			1	0.53–2.34		
Completed COVID-19 vaccine primary series	1.5	0.77–2.90			1.37	0.32–5.91		
Received COVID-19 booster dose	**2.25**	**1.48–3.42** *	**2.49**	**1.47–4.20**	1.23	0.32–4.68		
Intention to receive annual COVID-19 vaccines in the future	*Not considered in bivariate or multivariable models*				**11.69**	**7.58–18.02** *	**9.64**	**4.37–21.28**
F-statistic (*p*-value)	31.48 (<0.0001)				24.49 (<0.0001)			

* Considered for inclusion in multivariable model.

† Self-reported; including obesity, diabetes, heart disease, lung disease, immunocompromising condition, and other.

**Table 7:** Factors associated with COVID-19 vaccination uptake and intended uptake among health workers—Lao PDR, November 2023.

Variable	Received a COVID-19 booster dose				Intention to receive annual COVID-19 booster doses, if recommended by WHO			

	OR	95 % CI	aOR (*N* = 1158)	95 % CI	OR	95 % CI	aOR (*N* = 1223)	95 % CI

Age (for each 10-year increase in age)	**2.08**	**1.54–2.82** *	**1.92**	**1.38–2.70**	**1.56**	**1.19–2.05** *	**1.51**	**1.03–2.22**
Female	0.85	0.40–1.79*	0.81	0.40–1.65	1.60	0.72–3.55*	1.48	0.68–3.21
Occupation								
Doctor	Ref.				Ref.			
Assistant doctor	1.17	0.28–4.89*			**3.73**	**1.03–13.52** *	**4.92**	**1.53–15.82**
Nurse	**0.48**	**0.27–0.83** *			1.32	0.87–2.01*	1.22	0.85–1.76
Assistant nurse	3.66	0.22–62.17*			**0.36**	**0.19–0.67** *	**0.42**	**0.22–0.83**
Midwife	1.04	0.31–3.50*			**3.26**	**1.14–9.30** *	**3.13**	**1.43–6.85**
Other	0.65	0.20–2.19*			**2.62**	**1.17–6.41** *	**2.59**	**1.16–5.94**
Health facility level								
Central hospital	Ref.				Ref.			
Provincial hospital	0.81	0.43–1.51			**1.78**	**1.04–3.04** *	1.61	0.91–2.86
District hospital	1.33	0.66–2.69			**2.81**	**1.87–4.21** *	**2.22**	**1.53–3.23**
Health center	1.46	0.51–4.23			**5.54**	**1.58–19.44** *	**5.24**	**1.46–18.87**
Years in healthcare	**1.09**	**1.05–1.14** *			1.04	1.01–1.07*		
Presence of any underlying chronic conditions^†^	2.29	0.95–5.55*			0.79	0.45–1.39		
Ever examined or diagnosed a patient with COVID-19	1.66	0.83–3.32*			0.82	0.52–1.29		
Ever treated a patient with a life-threatening complication due to COVID-19	1.28	0.77–2.12*			0.86	0.64–1.16		
Completed COVID-19 vaccine primary series	*Not considered in bivariate or multivariable models*				1.27	0.54–2.95		
Received COVID-19 vaccine booster dose	*Not considered in bivariate or multivariable models*				1.41	0.81–2.44*		
Received influenza vaccine prior to start of COVID-19 pandemic	1.11	0.70–1.75			**2.15**	**1.20–3.86** *		
Received an influenza vaccine in the last season	**2.68**	**1.49–4.54** *	**2.19**	**1.29–3.72**	**3.10**	**1.96–4.90** *	**2.51**	**1.24–5.09**
F-statistic (p-value)	19.60 (<0.0001)				8.94 (<0.0001)			

* Considered for inclusion in multivariable model.

† Self-reported; including obesity, diabetes, heart disease, lung disease, immunocompromising condition, and other.

**Table 8:** Factors associated with the intention to recommend influenza vaccination to patients—Lao PDR, November 2023.

Variable	OR	95 % CI	aOR (N = 1217)	95 % CI

Age (for each 10-year increase in age)	1.36	0.56–3.29*	0.97	0.45–2.12
Female	1.41	0.48–4.13*	1.52	0.51–4.56
Occupation				
Doctor	Ref.			
Assistant doctor	3.87	0.30–50.28		
Nurse	1.81	0.27–12.13		
Assistant nurse	0.72	0.02–22.09		
Midwife	1.11	0.08–16.27		
Other	0.41	0.07–2.46		
Years in healthcare	1.04	0.95–1.12		
Presence of any underlying chronic conditions^†^	1.65	0.37–7.32		
Health facility level				
Central hospital	Ref.			
Provincial hospital	0.88	0.23–3.28		
District hospital	0.93	0.23–3.81		
Health center	*Removed from model (perfectly predicts outcome)*			
Ever examined or diagnosed a patient with influenza	**6.57**	**1.73–24.94** *	**6.69**	**1.72–26.02**
Ever treated a patient with a life-threatening complication due to influenza	**5.21**	**1.23–22.03** *		
Received influenza vaccine prior to start of COVID-19 pandemic	3.07	0.71–13.32*		
Received influenza vaccine in the last season	0.86	0.25–2.96*		
Intention to receive influenza vaccine in the future	**9.8**	**2.59–37.09** *		
Do you recommend COVID-19 vaccination to patients?	**13.23**	**2.01–87.25** *		
Intention to recommend annual COVID-19 vaccine to patients	**22.02**	**5.87–82.59** *	**21.41**	**5.34–85.91**
F-statistic (p-value)	11.57 (<0.0001)			

* Considered for inclusion in multivariable model.

† Self-reported; including obesity, diabetes, heart disease, lung disease, immunocompromising condition, and other.

**Table 9:** Factors associated with the intention to recommend annual COVID-19 vaccination to patients—Lao PDR, November 2023.

Variable	OR	95 % CI	aOR (N = 1228)	95 % CI

Age (for each 10-year increase in age)	**2.12**	**1.31–3.43** *	1.68	0.99–2.84
Female	1.45	0.63–3.30*	1.14	0.49–2.63
Occupation				
Doctor	Ref.			
Assistant doctor	0.53	0.14–1.95*		
Nurse	1.96	0.90–4.27*		
Assistant nurse	2.72	0.21–35.28*		
Midwife	**5.5**	**1.07–28.42** *		
Other	1.7	0.73–3.98*		
Years in healthcare	**1.08**	**1.02–1.14** *		
Presence of any underlying chronic conditions^†^	0.54	0.18–1.63		
Health facility level				
Central hospital	Ref.			
Provincial hospital	1.36	0.73–2.55*		
District hospital	**2.06**	**1.07–3.94** *		
Health center	*Removed from model (perfectly predicts outcome)*			
Ever examined or diagnosed a patient with COVID-19	0.91	0.23–3.65		
Ever treated a patient with a life-threatening complication due to COVID-19	1.54	0.54–4.36		
Received COVID-19 vaccine booster dose	3.79	**1.50–9.62** *		
Intention to receive annual COVID-19 vaccines in the future	28.69	**13.07–62.97** *	**24.73**	**11.43–53.53**
Intention to recommend influenza vaccine to patients	22.02	**5.87–82.59** *	**21.09**	**5.28–84.26**
F-statistic (p-value)	23.18 (<0.0001)			

* Considered for inclusion in multivariable model

† Self-reported; including obesity, diabetes, heart disease, lung disease, immunocompromising condition, and other

## Data Availability

Data will be made available on request.
